# Analytical Study for the Charge-Transfer Complexes of Rosuvastatin Calcium with π-Acceptors

**DOI:** 10.3390/molecules18077711

**Published:** 2013-07-03

**Authors:** Nourah Z. Alzoman, Maha A. Sultan, Hadir M. Maher, Mona M. Alshehri, Tanveer A. Wani, Ibrahim A. Darwish

**Affiliations:** 1 Department of Pharmaceutical Chemistry, College of Pharmacy, King Saud University, P.O. Box 2457, Riyadh 11451, Saudi Arabia; E-Mails: nalzoman@ksu.edu.sa (N.Z.A.); mahasultan59@gmail.com (M.A.S.); hadirrona@yahoo.com (H.M.M.); abs.office1@yahoo.com (M.M.A.); tanykash@yahoo.co.in (T.A.W.); 2 Department of Pharmaceutical Analytical Chemistry, Faculty of Pharmacy, University of Alexandria, El-Messalah, Alexandria 21521, Egypt

**Keywords:** atherosclerosis, cholesterol, rosuvastatin calcium, charge-transfer complexes, spectrophotometry, pharmaceutical analysis

## Abstract

Studies were carried out to investigate the charge-transfer (CT) reaction of ROS-Ca, as a n-electron donor with various π-acceptors: tetracyanoethylene, *p*-chloranilic acid, 2,3-dichloro-5,6-dicyano-1,4-benzoquinone, 2,3,5,6-tetrabromo-1,4-benzoquinone, 1,3,5-trinitrobenzene, 2,3,5,6-tetrachloro-1,4-benzoquinone, 7,7,8,8-tetracyano-quinodimethane, and 2,4,7-trinitro-9-fluorenone. Different colored CT complexes were obtained. The reaction mechanism and site of interaction were determined by ultraviolet-visible spectrophotometric techniques and computational molecular modeling. The formation of the colored complexes was utilized in the development of simple, rapid and accurate spectrophotometric methods for the determination of ROS-Ca. Under the optimum reaction conditions, linear relationships with good correlation coefficients (0.9984–0.9995) were found between the absorbances and the concentrations of ROS-Ca in the range of 2–200 μg mL^−1^. The limits of detection ranged from 0.41 to 12.24 μg mL^−1^. No interference could be observed from the additives commonly present in the tablets or from the drugs that are co-formulated with ROS-Ca in its combined formulations. The methods were successfully applied to the analysis of tablets with good accuracy and precision; the recovery percentages ranged from 99.54–100.46 ± 1.58–1.82%. The results were compared favorably with the reported method. The proposed methods are practical and valuable for routine application in quality control laboratories for determination of ROS-Ca in its bulk form and tablets.

## 1. Introduction

**Figure 1:**
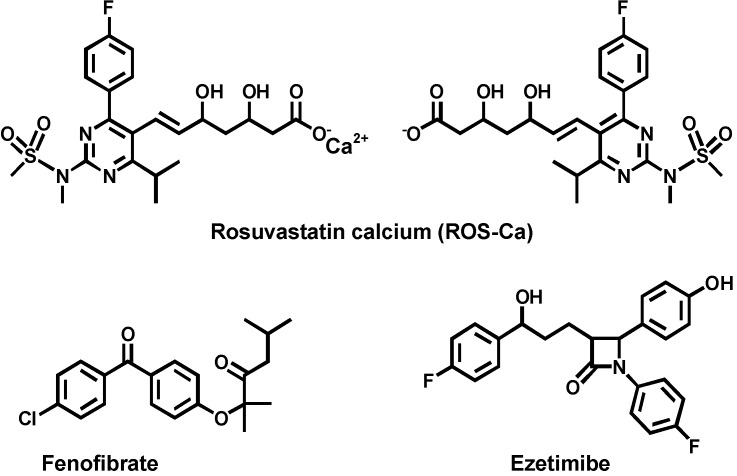
Chemical structures of rosuvastatin calcium (ROS-Ca) and the co-formulated drugs.

Rosuvastatin calcium (ROS-Ca, bis[(*E*)-7-[4-(4-fluorophenyl)-6-isopropyl-2-[methyl(methyl- sulfonyl)-amino] pyrimidin-5-yl](3*R*,5*S*)-3,5-dihydroxyhept-6-enoic acid calcium salt, Figure 1) is a synthetic 3-hydroxy-3-methylglutaryl-coenzyme A (HMG-CoA) reductase inhibitor. It exerts its action by specifically inhibiting the HMG-CoA reductase, the enzyme that catalyzes the conversion of HMG-CoA to mevolanate, which is the early rate-limiting step in the biosynthesis of cholesterol in the body. Inhibition of the enzyme decreases de novo cholesterol synthesis, increasing expression of low-density lipoprotein (LDL) receptors on hepatocytes. This increases the uptake of LDL by the hepatocytes, decreasing the amount of LDL-cholesterol in the blood. ROS-Ca also reduces blood levels of triglycerides and slightly increases levels of HDL-cholesterol [1,2,3].

The therapeutic importance of ROS-Ca was behind the growing interest in the development of analytical methods for its determination in its bulk and pharmaceutical dosage forms. A literature survey revealed that several analytical methods were reported for ROS-Ca determination. These methods include high-performance thin-layer chromatography [4], High performance liquid chromatography (HPLC) [5,6], and capillary zone electrophoresis [7].

HPLC is an efficient analytical technique and it is widely applied in pharmaceutical analysis; however it relies on expensive instrumentation that is not available in most quality control laboratories and time-consuming procedures in establishing the most appropriate chromatographic conditions. In general, spectrophotometry is the most widely used technique in pharmaceutical analysis because of its inherent simplicity and wide availability in most quality control laboratories [8,9,10,11,12,13]. For these reasons, spectrophotometric method for determination of ROS-Ca is required as an alternative for HPLC. The spectrophotometric methods that have been reported for determination of ROS-Ca suffer from major drawbacks [14,15,16,17]. These drawbacks include decreased selectivity due to measuring the native light absorption of ROS-Ca in the blue-shifted ultraviolet region, which might be subjected to interferences, employment of multiple-steps of non-selective oxidation reactions and tedious liquid-liquid extraction procedures using large volumes of organic solvents in the methods based on formation of ion-pair associates. Therefore, the development of new alternative spectrophotometric methods for determination of ROS-Ca in its bulk form and pharmaceutical dosage forms (tablets) is very essential.

The molecular interactions between the electron-donating pharmaceutical compounds and electron-accepting reagents are generally associated with the formation of intensely colored CT complexes, which usually absorb radiations in the visible region. The rapid formation of these complexes leads to their widespread utility in the development of visible spectrophotometric methods for analysis of many pharmaceutical compounds [18,19,20,21,22,23,24,25]. Literature survey revealed that the CT reaction of ROS-Ca has not been investigated yet. This fact promoted our interest in employment of the CT-reaction as a basis for the development of new spectrophotometric methods for determination of ROS-Ca.

## 2. Results and Discussion

### 2.1. Spectral Characteristics of the Reaction

**Figure 2:**
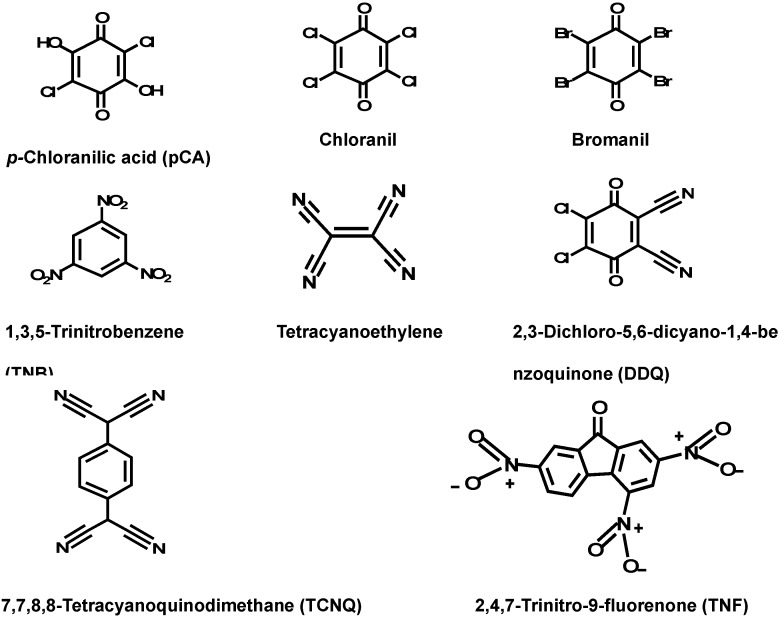
Chemical structures of the polyhaloquinone and polycyanoquinone π-acceptors used in the present study.

The interaction of ROS-Ca (Figure 1) with polyhaloquinone and polycyanoquinone π-acceptors (Figure 2) in non-polar solvents such as dichloroethane was found to produce colored CT complexes with low molar absorptivity values. 

In polar solvents such as methanol or acetonitrile, complete electron transfer from the ROS-Ca (D), as an electron donor, to the acceptor moiety (A) takes place with the formation of intensely colored radical ions with high molar absorptivity values, according to the following scheme:

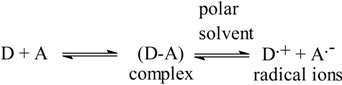



The dissociation of the (D-A) complex was promoted by the high ionizing power of the polar solvent and the resulting peaks in the absorption spectra of ROS-Ca-acceptor reaction mixtures were similar to the maxima of the radical anions of the acceptors obtained by the iodide reduction method [26].

**Figure 3:**
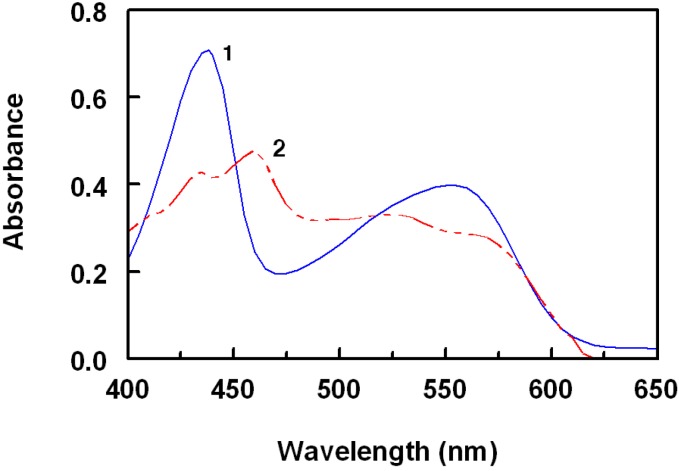
Absorption spectra of the CT complexes of ROS-Ca with TNB (1) and DDQ (2) Concentrations of ROS-Ca were 50 and 15 μg mL^−1^ in case of TNB and DDQ, respectively. Solutions were prepared in acetonitrile for reaction with TNB and in methanol for reaction with DDQ.

**Figure 4:**
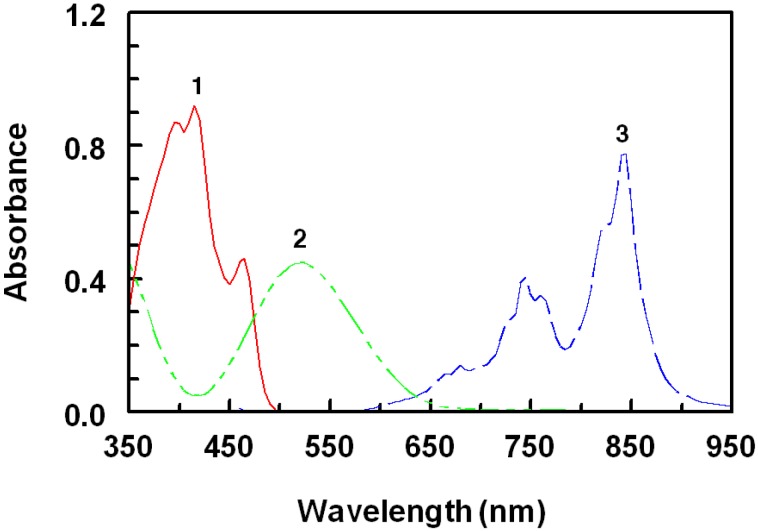
Absorption spectra of the CT complexes of ROS-Ca with TCNE (1), pCA (2), and TCNQ (3). Concentrations of ROS-Ca were 50, 70, and 20 μg mL^−1^ in case of TCNE, pCA, and TCNQ, respectively. Solutions were prepared in acetonitrile for reaction with all acceptors.

**Figure 5:**
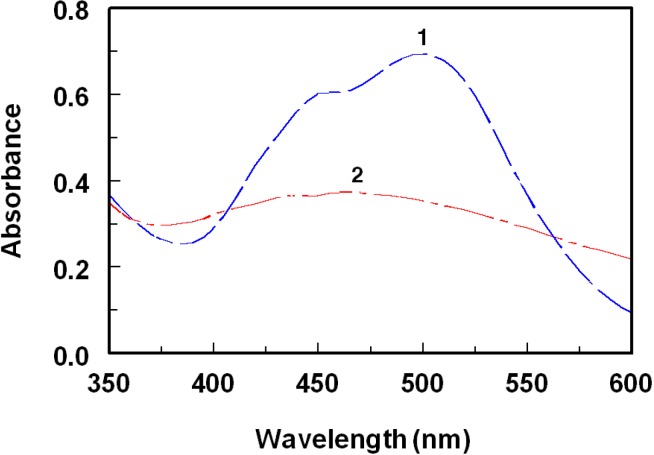
Absorption spectra of the CT complexes of ROS-Ca with bromanil (1) and chloranil (2). Concentrations of ROS-Ca were 80, and 150 μg mL^−1^ in case of bromanil and chloranil, respectively. Solutions were prepared in acetonitrile for reaction with both acceptors.

The interaction of ROS-Ca with π-acceptors at room temperature gave colored chromogens showing different absorption maxima at 840, 435, 460, 518, 412, 498, 460, and 412 nm for TCNQ, TNB, DDQ, pCA, TCNE, bromanil, chloranil, and TNF, respectively (Figure 3, Figure 4, Figure 5).

The predominant chromogen with TCNQ in acetonitrile is the bluish-green colored radical anion, which exhibits strong absorption maxima at 840, 823, 760, and 740 nm (Figure 4). These bands may be attributed to the formation of the radical anion TCNQ^−^, which was probably formed by the dissociation of an original donor-acceptor (D-A) complex with ROS-Ca. The dissociation of the complex was promoted by the high ionizing power of acetonitrile. Further support of this assignment was provided by the absorption maxima with those of TCNQ radical anion produced by the iodide reduction method [26]. 

The complex of ROS-Ca with TNB showed two absorption maxima at 435 and 550 nm (Figure 3). The intensity of the first maximum is about 1.5 fold the second one. Therefore, measurements were carried out at 435 nm, at which higher sensitivity was achieved. 

Chloranilic acid (pCA) exists in three ionic forms, the neutral yellow-orange H_2_A at very low pH, the dark purple HA^−^ which is stable at pH = 3 and a pale violet A^2−^, which is stable at high pH; these transformations are illustrated in the following scheme:

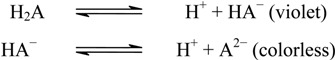



Since the interaction of ROS-Ca with pCA in acetonitrile gave a violet product (Figure 4), it might be concluded that HA was the form of pCA involved in the reaction described herein.

With TCNE, the characteristic shaped absorption band of TCNE radical anion with reported maximum in acetonitrile at 430 nm was not found (Figure 4). Instead, a duplet at 392 and 412 nm was formed which corresponds to the 1,2,3,3-pentacyanopropeneide (PCNP) anion, which is more preferable than TCNE anion, in quantitative analysis, in having higher molar absorptivity [26]. The resulting maxima of ROS-Ca with DDQ (Figure 3), bromanil, chloranil (Figure 5), and TNF are similar to that of radical anions of these acceptors obtained by the reduction method and coincide with the values reported in the literature [27,28].

The relative sensitivity of the eight acceptors employed in the present analytical work may be attributed to their difference in electron affinities, as well as the conditions employed in the reaction (reagent concentration, reaction time, and solvent). Bromanil, chloranil, and TNF gave relatively weak molar absorptivity values (Table 1). This may be explained on the basis of insufficient ionization of these relatively weak π-acceptors that possess lower electron affinities than TCNQ and DDQ [29]. Because of the poor sensitivity of TNF and its very slow reaction, it has been excluded from further investigations.

### 2.2. Optimization of Reaction Conditions

The results of variations in the reagents concentrations indicated that 1 mL of the concentrations indicated in Table 1 were the optimum concentrations. The higher concentrations of the reagents may be useful for rapidly reaching equilibrium, thus minimizing the time required to attain maximum absorbance at the corresponding wavelengths of maximum absorbance.

In order to select the most appropriate solvent, the reactions were carried out in different solvents. Small shifts in the position of the maximum absorption peak were observed, and the absorption intensities were also influenced. Methanol gave maximum sensitivity in the case of DDQ and acetonitrile was considered as an ideal solvent for the other acceptors. This because it offered maximum sensitivity, which was attributed to the high dielectric constant of acetonitrile that promotes maximum yield of radical anions, in addition to its high solvating power for the acceptors [30].

The optimum reaction time was determined by monitoring the color development at room temperature (25 ± 2 °C). Complete color development was attained instantaneously with DDQ, and PCA, or after 5–60 min with other acceptors (Table 1). The developed colors remained stable at room temperature for at least a further 30 min. 

### 2.3. Molar Ratio of the Reaction, Molecular Modeling, and Proposing the Site of Interaction

**Figure 6:**
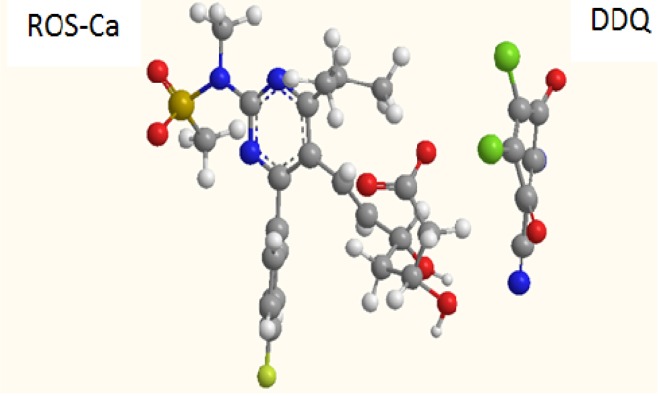
Energy-minimized CT complex of ROS-Ca with DDQ.

Job’s method of continuous variation was used for determining the molar ratio of ROS-Ca to DDQ. From the obtained Job’s plot (data not shown), it was concluded that the ROS-Ca:DDQ ratio is 1:2. This indicated that two moles of DDQ interacted with one mole of ROS-Ca. Considering the divalent calcium ion, the reaction was postulated to proceed as 1:1 ratio for DDQ with rosuvastatin anion via only one site of interaction in spite of the presence of more than one possible electron-donating site. For investigating the site of interaction and postulate the reaction mechanism, modeling for the CT complex was performed. It was found that the highest electron densities in the rosuvastatin molecule are located on the two oxygen atoms of the sulfonamide group. The total charges on each of the two oxygen atoms of the sulfonamide anion were found to be −0.96543 and −0.97147. As well, it was found that DDQ moves toward the sulfonamide group of ROS to form the CT complex (Figure 6). In the sulfonamide group, sulfur can donate a major share of the lone pairs of electrons to the two oxygen atoms. This leads to the development of partial negative charges on the oxygen atoms, and this makes them capable of forming the charge transfer complex. Other centers did not contribute in the CT reaction based on the fact that certain electron density was required for achievement of a successful electron transfer [29].

**Table 1:** Optimum conditions for the CT reaction of ROS-Ca with different π-acceptors and the achieved molar absorptivities.

Acceptor ^a^	Condition				Molar absorptivity (ε × 10^−4^)
	Reagent conc. (mg mL^−1^)	Solvent	Time (min)	λ_max_ (nm)	
pCA	4	Acetonitrile	At once ^b^	518	1.4
DDQ (1.9)	2	Methanol	At once ^b^	460	3.0
TCNE (2.2)	2	Acetonitrile	15	412	1.8
TNB (0.7)	4	Acetonitrile	30	435	0.64
TCNQ (1.7)	1	Acetonitrile	15	840	4.0
Bromanil (1.37)	5	Acetonitrile	5	498	0.88
Chloranil (1.37)	5	Acetonitrile	5	460	2.2
TNF (1.1)	5	Acetonitrile	60	412	0.25

^a^ Figures in parenthesis are the electron affinities of the acceptors [29]. Electron affinity of pCA could not be found in the available literatures. ^b^ For getting readings with high precision, the reactions were allowed to proceed for 5 min.

### 2.4. Development and Validation of the Analytical Methods

#### 2.4.1. Calibration Curves, Linearity and Sensitivity

Under the specified optimum reaction conditions, the calibration curves for ROS-Ca with the different analytical reagents employed in the present work were constructed. The regression equations for the results were derived using the least-squares method. In all cases, Beer’s law plots (n = 5) were linear with very small intercepts and good correlation coefficients in the general concentration range of 2–200 μg mL^−1^ (Table 2). The limits of detection (LOD) and limits of quantitation (LOQ) were determined [31] using the formula: LOD or LOQ = κSDa/b, where κ = 3 for LOD and 10 for LOQ, SDa is the standard deviation of the intercept, and b is the slope. Based on the basis of 3 replicate measurements, the limits of detection were 0.41–12.24 μg mL^−1^.

#### 2.4.2. Precision

The precisions of the assays (within-assay and between-assays) were determined at the ROS-Ca concentrations cited in Table 3. The within-assay precision was assessed by analyzing six replicates of each sample as a batch in a single assay run, and the between-assays precision was assessed by analyzing the same sample, as triplicate, in two separate assay runs. The assays gave satisfactory results; the relative standard deviations (RSD) were less than 2% (Table 3). This level of precision of the proposed methods was adequate for the quality control analysis of ROS-Ca. 

#### 2.4.3. Specificity and Interference

The proposed assay has the advantages that the measurements are performed in the visible region, away from the UV-absorbing interfering substances that might be co-extracted from dosage forms that contain ROS-Ca. The interference from the congenital drugs that is co-formulated with ROS-Ca in some of its dosage forms was studied. These drugs were fenofibrate [32] and ezetimibe [33]. The chemical structures of these drugs are given in Figure 1. Potential interferences of these drugs were studied in a ratio which is normally present in their combined dosage forms. No interferences from these drugs were found with ROS-Ca in the proposed assay. This selectivity of the CT reaction for ROS-Ca was attributed to its adequate basic character (electron-donating), which allowed the CT, rather than the other drug, that does not have the adequate basic property that is required to achieve CT reaction. As well, no interference was observed from the excipients with the proposed assay as indicated from the obtained good recovery (mentioned above). The absence of interference from the excipients, even though they contain basic component(s) was attributed to the extraction of the ROS-Ca tablets prior to the analysis with methanol in which the excipients do not dissolve.

**Table 2:** Quantitative parameters for the determination of ROS-Ca based on its CT reaction with various π-acceptors.

Acceptor	Range (μg mL^−1^)	Intercept	Slope	Correlation coefficient	LOD (μg mL^−1^)	LOQ (μg mL^−1^)
TCNQ	5–50	0.0032	0.0388	0.9995	0.41	1.37
TNB	4–30	0.0171	0.0137	0.9992	1.52	5.07
DDQ	2–40	0.0146	0.0304	0.9990	1.14	3.80
pCA	10–150	0.0211	0.0061	0.9987	1.82	6.07
TCNE	5–60	0.0062	0.0183	0.9989	4.32	14.39
Bromanil	25–100	0.0074	0.0087	0.9993	5.11	17.02
Chloranil	40–200	0.0171	0.0021	0.9984	12.24	40.76

**Table 3:** Precision of the proposed methods for determination of ROS-Ca based on its CT reaction with different acceptors.

Acceptor-based method	ROS-Ca (μg mL^−1^)	Within-assay, n = 6		Between-assays, n = 6	
		Mean (μg mL ^−1^ ± SD)	RSD	Mean (μg mL ^−1^ ± SD)	RSD
pCA	50	48.78 ± 0.41	0.84	50.05 ± 0.84	1.68
DDQ	20	20.51 ± 0.25	1.22	19.56 ± 0.36	1.84
TCNE	40	40.57 ± 0.75	1.85	38.96 ± 0.75	1.93
TNB	20	20.73 ± 0.23	1.11	19.55 ± 0.35	1.79
TCNQ	40	38.78 ± 0.16	0.41	38.04 ± 0.29	0.76
Bromanil	80	81.57 ± 0.84	1.03	78.24 ± 0.81	1.04
Chloranil	200	198.78 ± 2.69	1.35	201.05 ± 2.08	1.03

#### 2.4.4. Ruggedness and Robustness

The ruggedness of the proposed methods was assessed by applying the procedures using two different instruments (shown in the Experimental section) in two different laboratories at different elapsed time. Results obtained from lab-to-lab and day-to-day were found to be reproducible as RSD did not exceed 2%. Robustness of the procedures was assessed by evaluating the influence of small variation of experimental variables: concentrations of acceptor reagent, and reaction time, on the analytical performance of the method. In these experiments, one experimental parameter was changed while the other parameters were kept unchanged, and the recovery percentage was calculated each time. The small variations in any of the variables did not significantly affect the results; recovery percentages were 97.25–103.51% ± 0.74–1.92%. This provided an indication for the reliability of the proposed methods during routine work. 

### 2.5. Application of the Method to the Analysis of Tablets

The obtained satisfactory validation results made the proposed procedures suitable for the routine quality control analysis of ROS-Ca. The proposed and the reported method [14] were applied to the determination of ROS-Ca in its tablets. The results obtained by the proposed methods were statistically compared with those obtained by the reported method. The obtained values of the labeled amount were 99.54–100.46 ± 1.58–1.82% (Table 4). In the *t-* and F-tests, no significant differences were found between the calculated and theoretical values of both the proposed and the reported methods at 95% confidence level. This indicated similar precision and accuracy in the analysis of ROS-Ca in its capsules. It is evident from these results that all the proposed methods are applicable to the analysis of ROS-Ca in its pharmaceutical tablets with comparable analytical performance. However, the critical recommendations of some of these methods might be based on the experimental conditions (e.g., reaction time), and the ultimate sensitivity that determines the amount of specimen required for analysis. For example, the methods involving DDQ, pCA, bromanil, and chloranil are recommended whenever rapid analysis is required; this because they have very short reaction time. The method involving TCNQ is recommended, as high sensitivity is required on the expense of the analysis time. 

**Table 4:** Determination of ROS-Ca in its tablets by the proposed methods.

Method	Label claim (% ± SD) ^a^	t-values ^b^	F-values ^b^
pCA	99.58 ± 1.77	1.55	2.41
DDQ	100.21 ± 1.43	1.26	1.57
TCNE	99.54 ± 1.58	1.86	1.92
TNB	99.82 ± 0.78	0.83	0.47
TCNQ	100.09 ± 1.96	0.61	2.20
Bromanil	100.29 ± 1.32	1.72	1.34
Chloranil	100.46 ± 1.82	2.10	2.55
Reported ^c^	99.95 ± 1.14		

^a^ Values are mean of five determinations. ^b^ The tabulated values of t and F at 95% confidence limit are 2.78 and 6.39, respectively. ^c^ [14].

## 3. Experimental Section

### 3.1. Apparatus

Double beam ultraviolet-visible spectrophotometer with matched 1-cm quartz cells (UV-1601 PC; Shimadzu, Kyoto, Japan) was used for all the spectrophotometric measurements. 

### 3.2. Chemicals and Reagents

ROS-Ca was obtained from Pfizer Inc. (New York, NY, USA). Ezetimibe was obtained from AK Scientific Inc. (30023 Ahern Avenue Union City, CA, USA). Fenofibrate was obtained from Sigma Chemical Co. (St. Louis, MO, USA). The purities of the investigated compounds were >99%, and the solutions were stable for at least one week when kept refrigerated). 7,7,8,8-tetracyanoquinodimethane (TCNQ; Aldrich Chem. Co, Milwaukee, WI, USA) was 1 mg mL^−1^ in acetonitrile, and the solution was stable for at least 1 week at 4 °C. 1,3,5-Trinitrobenzene (TNB; Prodotti Chimiol Pet Analist Scientifico, Milano, Italy) was 4 mg mL^−1^ in acetonitrile, and the solution was prepared fresh daily. 2,3-Dichloro-5,6-dicyano-1,4-benzoquinone (DDQ; Merck Schuchardt, Munich, Germany) was 2 mg mL^−1^ in methanol and it was prepared fresh daily. 2,5-Dichloro-3,6-dihydroxy-1,4-benzoquinone (chloranilic acid, pCA) from BDH Chemicals (Poole, UK) was 4 mg mL^−1^ in acetonitrile, and the solution was prepared fresh daily. Tetracyanoethylene (TCNE; Nacalai Tesque, Kyoto, Japan) was 1 mg mL^−1^ in acetonitrile, and it was prepared fresh daily. 2,3,5,6-Tetrabromo-1,4-benzoquinone (bromanil; Hopkin & Williams Ltd, Heneage Street, Birmingham, UK), and 2,3,5,6-tetrachloro-1,4-benzoquinone (chloranil; Sigma Chemical Co. 3050 Spruce St St Louis, MO 63103, USA) were 5 mg mL^−1^ in acetonitrile, and the solutions were prepared fresh daily. 2,4,7-Trinitro-9-fluorenone (TNF; Fluka, Buchs, Switzerland) was 5 mg mL^−1^ in acetonitrile and the solution was prepared fresh daily. Crestor tablets (Astra Zeneca Pharma, Bengaluru, India) labeled to contain 5 mg ROS-Ca were obtained from the market. All solvents and other chemicals used throughout this study were of analytical grade.

### 3.3. Preparation of Standard and Tablets Sample Solutions

#### 3.3.1. Preparation of Stock Standard ROS-Ca Solution

Into a 50-mL calibrated flask, ROS-Ca (100 mg) was accurately weighed and dissolved in methanol (2 mL), completed to volume with methanol for DDQ and with acetonitrile for the other acceptors. This stock solution (2 mg mL^−1^) was diluted with the respective solvents to obtain suitable concentrations that lie in the linear range of each particular assay method.

#### 3.3.2. Preparation of Tablets Sample Solution

Twenty tablets were weighed and finely powdered. A quantity of the powder equivalent to 50 mg of ROS-Ca was transferred into a 25-mL calibrated flask, dissolved in methanol (2 mL), swirled and sonicated for 5 min, completed to volume with the corresponding solvent (as in stock solution), shaken well for 15 min, and filtered. The first portion of the filtrate was rejected, and a measured volume of the filtrate was diluted quantitatively with a suitable solvent to yield suitable concentrations as the linear range of each particular assay method. 

### 3.4. General Analytical Procedure

One milliliter of the standard or sample solution of ROS-Ca (20–2,000 μg mL^−1^) was transferred into 10-mL calibrated flasks. One milliliter of the acceptor solution was added, and the reaction was allowed to proceed at room temperature (25 ± 2 °C) for 5 min (in case of pCA, DDQ, bromanil and chloranil), 15 min (in case of TCNQ and TCNE), 30 min (in case of TNB), and for 60 min (in case of TNF). The solutions were diluted to volume with methanol for DDQ and with acetonitrile for the other acceptors. The absorbances of the resulting solutions were measured at the wavelengths of maximum absorption (840, 435, 460, 518, 412, 498, 460, and 412 nm for TCNQ, TNB, DDQ, pCA, TCNE, bromanil, chloranil, and TNF, respectively) against reagent blanks treated similarly. 

### 3.5. Determination of Molar Ratio

The Job’s method of continuous variation [34] was employed. Master equimolar solutions of ROS-Ca and reagents were prepared. The concentrations of these solutions were 4.9 × 10^−3^ M (in acetonitrile for TCNQ), 1.9 × 10^−2^ M (in acetonitrile for TNB), 8.8 × 10^−3^ M (in methanol for DDQ), 1.9 × 10^−2^ M (in acetonitrile for pCA), 1.6 × 10^−2^ M (in acetonitrile for TCNE), 1.2 × 10^−2^ M (in acetonitrile for bromanil), and 2 × 10^−2^ M (in acetonitrile for chloranil). Series of 10-ml portions of the master solutions of ROS-Ca with the respective reagent were made up comprising different complementary proportions (0:10, 1:9, ………, 9:1, 10:0, inclusive) in 10-mL calibrated flasks. The reactions were allowed to proceed for the optimum reaction time (Table 1) and then the absorbances of the resulting solutions were measured at the corresponding wavelengths of maximum absorbances (λ_max_).

### 3.6. Molecular Modeling for the CT Complex of ROS-Ca with DDQ

The molecular modeling for the CT complex of ROS-Ca with DDQ was performed by using CS Chem3D Ultra, version 9 (Cambridge Soft Corporation, Cambridge, MA, USA) implemented with molecular orbital computations software (MOPAC), and molecular dynamics computations software.

## 4. Conclusions

The CT reaction of ROS-Ca as electron donor and some p-electron acceptors has been investigated. The obtained complexes were studied by ultraviolet-visible spectrophotometry. The obtained colored CT complexes were utilized in the development of seven simple, rapid and accurate spectrophotometric methods for the analysis of ROS-Ca in pure form as well as in capsules. The methods described herein have many advantages: they do not need expensive sophisticated apparatus, they are simple and rapid, and they have high sensitivity. The proposed methods used inexpensive reagents with excellent shelf life, and are available in any analytical laboratories. Therefore, the methods are practical and valuable for routine application in quality control laboratories for analysis of ROS-Ca.

## References

[1] Cheng-Lai A. (2003). Rosuvastatin: A new HMG-CoA reductase inhibitor for the treatment of hypercholesterolemia. Heart Dis..

[2] Cheng J.W. (2004). Rosuvastatin in the management of hyperlipidemia. Clin. Ther..

[3] Wani T.A., Samad A., Tandon M., Saini G.S., Sharma P.L., Pillai K.K. (2010). The effects of rosuvastatin on the serum cortisol, serum lipid, and serum mevalonic acid levels in the healthy Indian male population. AAPS Pharm. Sci. Tech..

[4] Chaudhari B.G., Patel N.M., Shah P.B. (2007). Determination of simvastatin, pravastatin sodium and rosuvastatin calcium in tablet dosage forms by HPTLC. Indian J. Pharm. Sci..

[5] Kaila H.O., Ambasana M.A., Thakkar R.S., Saravaia H.T., Shah A.K. (2010). A new improved RP-HPLC method for assay of rosuvastatin calcium in tablets. Indian J. Pharm. Sci..

[6] Pandya C.B., Channabasavaraj K.P., Chudasama J.D., Mani T.T. (2010). Development and validation of RP-HPLC method for determination of rosuvastatin calcium in bulk and pharmaceutical dosage form. Inter. J. Pharm. Sci. Rev. Res..

[7] Suslu I., Çelebier M., Altınoz S. (2007). Determination of rosuvastatin in pharmaceutical formulations by capillary zone electrophoresis. Chromatographia.

[8] Darwish I.A., Sultan M.A., Al-Arfaj H.A. (2009). Novel selective kinetic spectrophotometric method for determination of norfloxacin in its pharmaceutical formulations. Talanta..

[9] Darwish I.A., Abdine H.H., Amer S.M., Al-Rayes L.I. (2009). Spectrophotometric study for the reaction between fluvoxamine and 1,2-naphthoquinone-4-sulphonate: Kinetic, mechanism and use for determination of fluvoxamine in its dosage forms. Spectrochim. Acta. A.

[10] Darwish I.A., Hussein S.A., Mahmoud A.M., Hassan AI. (2008). Spectrophotometric determination of H(2)-receptor antagonists via their oxidation with cerium(IV). Spectrochim. Acta A.

[11] Ahmed S., Rasul A., Masood Z. (2011). Spectrophotometry in Pharmaceutical Analysis.

[12] Gorog S. (1995). Ultraviolet-visible Spectrophotometry in Pharmaceutical Analysis.

[13] Darwish I.A., Refaat I.H., Askal H.F., Marzouq M.A. (2006). Generic nonextractive spectrophotometric method for determination of 4-quinolone antibiotics by formation of ion-pair complexes with beta-naphthol. J. AOAC Int..

[14] Uyar B., Celebier M., Altinoz S. (2007). Spectrophotometric determination of rosuvastatin calcium in tablets. Pharmazie.

[15] Sevda R.R., Ravetkar A.S., Shirote P.J. (2011). UV Spectrophotometric estimation of rosuvastatin calcium and fenofibrate in bulk drug and dosage form using simultaneous equation method. Int. J. Chem. Tech. Res..

[16] Gupta A., Mishra P., Shah K. (2009). Simple UV spectrophotometric determination of rosuvastatin calcium in pure form and in pharmaceutical formulations. E-J. Chem..

[17] Marothu V.K., Dannana G.S. (2007). Extractive spectrophotometric methods for the determination of rosuvastatin calcium in pure form and in pharmaceutical formulations by using Safranin O and Methylene blue. E-J. Chem..

[18] Darwish I.A. (2005). Development and validation of spectrophotometric methods for determination of fluoxetine, sertraline, and paroxetine in pharmaceutical dosage forms. J. AOAC Int..

[19] Darwish I.A., Wani T.A., Khalil N.Y., Al-Shaikh A.A., Al-Morshadi N. (2012). Development of a novel 96-microwell assay with high throughput for determination of olmesartan medoxomil in its tablets. Chem. Cent. J..

[20] Wani T.A., Khalil N.Y., Abdel-Rahman H.M., Darwish I.A. (2011). Novel microwell-based spectrophotometric assay for determination of atorvastatin calcium in its pharmaceutical formulations. Chem. Cent. J..

[21] Darwish I.A. (2005). Kinetic spectrophotometric assays for determination of trimetazidine dihydrochloride. Anal. Chim. Acta.

[22] Darwish I.A. (2005). Analytical study for the charge transfer complexes of losartan potassium. Anal. Chim. Acta.

[23] Darwish I.A., Refaat I.H. (2006). Spectrophotometric analysis of selective serotonin reuptake inhibitors based on formation of charge-transfer complexes with tetracyanoquinodimethane and chloranilic acid. J. AOAC Int..

[24] Darwish I., Abdel-Wadood H., Abdel-Latif N. (2005). Validated spectrophotometric and fluorimetric methods for analysis of clozapine in tablets and urine. Ann. Chim..

[25] Huang W., Liu X.J., Zhao F.L. (2006). Spectrophotometric determination of azithromycin by charge transfer reaction. Chin. J. Mod. Appl. Pharm..

[26] Taha A., Rucker G. (1977). Utility of pi-acceptors in alkaloid assay. Arch. Pharm. (Weinheim.).

[27] Melby L.R. (1970). The Chemistry of the Cyano Group.

[28] Yamagishi A. (1975). Solvation effects on the electron-transfer reaction of TCNQ anion radical and 2,3-dichloro-5, 6-dicyano-p-benzoquinone. Bull. Soc. Jpn..

[29] Foster R. (1969). Organic Charge-Transfer Complexes.

[30] (1989). Vogel’s Textbook of Practical Organic Chemistry.

[31] (2008). The United States Pharmacopeia 24, The National Formulary 19.

[32] Agouridis A.P., Tsimihodimos V., Filippatos T.D., Tselepis A.D., Elisaf M.S. (2011). High doses of rosuvastatin are superior to low doses of rosuvastatin plus fenofibrate or n-3 fatty acids in mixed dyslipidemia. Lipids.

[33] Kawashiri M.A., Nohara A., Noguchi T., Tada H., Nakanishi C., Mori M., Konno T., Hayashi K., Fujino N., Inazu A. (2012). Efficacy and safety of coadministration of rosuvastatin, ezetimibe, and colestimide in heterozygous familial hypercholesterolemia. Am. J. Cardiol..

[34] Job P. (1964). Advanced Physicochemical Experiments.

